# Supplementary material for: Deep Learning Pose Estimation for Phenotyping of Co‐Occurring Hyperkinetic Movement Disorders
Source: Ann Clin Transl Neurol. 2026 Jul 25:10.1002/acn3.70474. Online ahead of print. doi: 10.1002/acn3.70474 (PMC13401409; doi:10.1002/acn3.70474)
Supplement: Supplementary file 2 — Supporting Information S2: Considerations related to phenomenological distinctions. [file ACN3-9999-0-s002.docx]

**Supplementary material 2**

### Methodological Considerations

**Phenomenological Distinctions**

CODY-SAMP addresses distinct MD patterns individually to enable precise phenotypic characterization. Ballismus is considered as part of the chorea spectrum and involves similar pathophysiologic mechanisms. Athetosis, characterized by slow, continuous, involuntary, sinuous movements of distal body parts preventing maintenance of stable posture, is most often observed in dyskinetic cerebral palsy and frequently accompanies dystonia. Its spatiotemporal characteristics make it appear on a continuum between dystonia and chorea (57). The term choreoathetosis is commonly used to describe movements difficult to classify as purely chorea or athetosis. CODY-SAMP addresses chorea, ballismus, and athetosis individually given possible differences in physiological backgrounds and therapeutic implications.

Tics and stereotypies are among the most common MDs in the neurodevelopmental period (58), and more frequent with intellectual and developmental disability, were rare in our samples. Differentiating between them can be challenging due to overlapping clinical features, however clinical history may be relevant given distinct treatment modalities (59).

**Subscale Design Rationale**

CODY_SAMP Subscales I and II were designed to assess dystonia and other hyperkinetic movement disorder while Subscale III captures associated neurological signs to further contribute to syndromic characterization. Subscale III items (parkinsonism, ataxia, UMN signs) are reported as present or absent without severity assessment, as other available scales address them in detail (60),(17),(14) and they are targeted by distinct therapeutic interventions. In children with neurodevelopmental, neurodegenerative, and neurometabolic disorders, hypotonia is frequent and may occur alone or associated with pediatric parkinsonism (60) or dystonia. We addressed these items individually to enable defining further syndromic associations. Ataxia shows age dependency in young patients (61) and we identified its presence without severity assessment. Tremor is included in dystonia definitions, with tremulous or jerky dystonia proposed in recent classification updates (8). However, CODY-SAMP reports tremor independently to capture and monitor different tremor types and distributions relevant to different tremor syndromic entities (1).

**Context of Pediatric Movement Disorder Assessment and Treatment**

Few rating scales for dystonia have been developed primarily for children (***Supplementary Table 1).*** The Barry-Albright Dystonia Scale was developed for assessing intrathecal baclofen outcomes in “secondary”, acquired dystonia (62). The Movement Disorder-Childhood Rating Scales offers two age-specific versions (0-3 years and 4-18 years) and demonstrates good construct validity and inter-rater reliability, but does not completely separate or discriminate among specific MDs (20). MD-CRS part II groups phenomenologically distinct disorders (hypokinetic-rigid syndrome, chorea/ballism, dystonia/athetosis), while other scales assess dystonia independently from chorea and athetosis (19), CODY-SAMP considers different HMDs as individual entities.

The most widely used scale remains the BFMDRS, designed for primary generalized dystonia and validated for measuring DBS clinical effects. However, no scale has been rigorously tested for validity across broad developmental ranges. Since most children with dystonia have other co-occurring MDs, valid methods to dissociate dystonia from other disorders in mixed presentations are essential (24) .

DBS may affect developing brains differently than mature brains (61). One potential DBS mechanism in dystonia involves alteration of abnormal neural plasticity. If DBS alters abnormal plasticity (63),(64)it may also alter normal plasticity. Monitoring clinical evolution with DBS across the lifespan is an unmet need, requiring properly designed clinical tools capable of capturing neuromodulation-related changes, whether therapeutical or potentially deleterious. The lack of universally valid rating scales and standardized methodologies for assessing dystonia combined with other MDs has limited research focus to specific disease entities, emphasizing the urgent need for valid and reliable instruments applicable across ages and mixed movement disorder presentations (24).

**References**

57. Yilmaz S, Mink JW. Treatment of Chorea in Childhood. Pediatr Neurol. 2020 Jan;102:10–9. doi:10.1016/j.pediatrneurol.2019.08.013

58. Zinner SH, Mink JW. Movement Disorders I: Tics and Stereotypies. Pediatr Rev. 2010 Jun 1;31(6):223–33. doi:10.1542/pir.31.6.223

59. Vermilion JA, Bitsko RH, Danielson ML, Bonifacio KP, Dean SL, Hyman SL, et al. Performance of a Tic Screening Tool (MOVeIT) in Comparison to Expert Clinician Assessment in a Developmental-Behavioral Pediatrics Clinic Sample. Evid-Based Pract Child Adolesc Ment Health. 2024;9(2):245–61. doi:10.1080/23794925.2023.2272948 PubMed PMID: 39109230; PubMed Central PMCID: PMC11299864.

60. Pons R, Pearson TS, Perez-Dueñas B, Garcia-Cazorla A, Kurian MA, Dalivigka Z, et al. Development and Preliminary Validation of a Parkinsonism-Dystonia Scale for Infants and Young Children. Mov Disord Off J Mov Disord Soc. 2025 Aug;40(8):1669–79. doi:10.1002/mds.30219 PubMed PMID: 40364572; PubMed Central PMCID: PMC12371672.

17. Goetz CG, Tilley BC, Shaftman SR, Stebbins GT, Fahn S, Martinez-Martin P, et al. Movement Disorder Society-sponsored revision of the Unified Parkinson’s Disease Rating Scale (MDS-UPDRS): scale presentation and clinimetric testing results. Mov Disord Off J Mov Disord Soc. 2008 Nov 15;23(15):2129–70. doi:10.1002/mds.22340 PubMed PMID: 19025984.

14. Schmitz-Hübsch T, Du Montcel ST, Baliko L, Berciano J, Boesch S, Depondt C, et al. Scale for the assessment and rating of ataxia: Development of a new clinical scale. Neurology. 2006 Jun 13;66(11):1717–20. doi:10.1212/01.wnl.0000219042.60538.92

61. Mink JW. The impact of development on the interpretation of movement disorders rating scales. Dev Med Child Neurol. 2014 Jun;56(6):511–2. doi:10.1111/dmcn.12464

8. Albanese A, Bhatia KP, Fung VSC, Hallett M, Jankovic J, Klein C, et al. Definition and Classification of Dystonia. Mov Disord. 2025 Jul;40(7):1248–59. doi:10.1002/mds.30220

1. Bhatia KP, Bain P, Bajaj N, Elble RJ, Hallett M, Louis ED, et al. Consensus Statement on the classification of tremors. from the task force on tremor of the International Parkinson and Movement Disorder Society. Mov Disord Off J Mov Disord Soc. 2018 Jan;33(1):75–87. doi:10.1002/mds.27121 PubMed PMID: 29193359; PubMed Central PMCID: PMC6530552.

62. Barry MJ, VanSwearingen JM, Albright AL. Reliability and responsiveness of the Barry–Albright Dystonia Scale. Dev Med Child Neurol. 1999 Jun;41(6):404–11. doi:10.1017/S0012162299000870

20. Battini R, Sgandurra G, Petacchi E, Guzzetta A, Di Pietro R, Giannini MT, et al. Movement Disorder-Childhood Rating Scale: Reliability and Validity. Pediatr Neurol. 2008 Oct;39(4):259–65. doi:10.1016/j.pediatrneurol.2008.07.002

19. Monbaliu E, Ortibus E, De Cat J, Dan B, Heyrman L, Prinzie P, et al. The Dyskinesia Impairment Scale: a new instrument to measure dystonia and choreoathetosis in dyskinetic cerebral palsy. Dev Med Child Neurol. 2012 Mar;54(3):278–83. doi:10.1111/j.1469-8749.2011.04209.x

24. Mink JW. Special concerns in defining, studying, and treating dystonia in children. Mov Disord Off J Mov Disord Soc. 2013 Jun 15;28(7):921–5. doi:10.1002/mds.25548 PubMed PMID: 23893449; PubMed Central PMCID: PMC3806453.

63. Ruge D, Cif L, Limousin P, Gonzalez V, Vasques X, Hariz MI, et al. Shaping reversibility? Long-term deep brain stimulation in dystonia: the relationship between effects on electrophysiology and clinical symptoms. Brain J Neurol. 2011 Jul;134(Pt 7):2106–15. doi:10.1093/brain/awr122 PubMed PMID: 21705425.

64. Cif L, Ruge D, Gonzalez V, Limousin P, Vasques X, Hariz MI, et al. The Influence of Deep Brain Stimulation Intensity and Duration on Symptoms Evolution in an OFF Stimulation Dystonia Study. Brain Stimulat. 2013 Jul;6(4):500–5. doi:10.1016/j.brs.2012.09.005
