# Supplementary material for: Deep Learning Pose Estimation for Phenotyping of Co‐Occurring Hyperkinetic Movement Disorders
Source: Ann Clin Transl Neurol. 2026 Jul 25:10.1002/acn3.70474. Online ahead of print. doi: 10.1002/acn3.70474 (PMC13401409; doi:10.1002/acn3.70474)
Supplement: Supplementary file 3 — Supporting Information S3: Computational methods. [file ACN3-9999-0-s005.docx]

# Supplementary Material S3 : Methods

## **(Table 1)** provides the major computational methods and tools employed across the end-to-end pipelines for video-based HMD classification. ^1–17 18^

**Table 1. Overview of Computational Methods, Libraries, and Challenges Identified in the Video-Based HMD Classification Pipeline.**

| **Method** | **Library** | **In literature** | **Goal** | **Effect** | **Problem** |
| --- | --- | --- | --- | --- | --- |
| Video loading / extraction | OpenCV (cv2) | Bradski, 2000 / Cao et al., 2019 | Load video files, extract metadata and frames | Enables frame-by-frame reproducible processing | Varying codecs, dropped frames |
| Frame-by-frame processing | OpenCV (cv2) | Bradski, 2000 / Cao et al., 2021 | Sequential access to each frame, maintain temporal order | Ensures precise temporal correspondence | Frame drops, timing jitter |
| 2D pose estimation | YOLOv8 (Ultralytics) | Redmon et al., 2016 / Jocher et al., 2023 / Mathis et al., 2018 | Detect human keypoints in each frame (pose estimation) | Fast, accurate localization of body landmarks | Occlusion, rare postures, lighting |
| Numerical feature extraction | NumPy, SciPy | Harris et al., 2020 / Virtanen et al., 2020 | Compute time series features: coordinates, distances, etc. | Enables quantitative description of motor behavior | Tracking noise, artifacts |
| Data structuring | pandas | McKinney, 2010 | Organize features and metadata in structured tables | Facilitates downstream analysis | Memory use with large datasets |
| Statistical feature engineering | pandas, NumPy, SciPy | Bandt & Pompe, 2002 / Harris et al., 2020 / Virtanen et al., 2020 | Summarize displacement signals: mean, std, median, etc. | Captures variability, trends, central tendency | Sensitive to outliers |
| Spectral feature extraction | SciPy (FFT) | Harris et al., 2020 / Zhang et al., 2022 / Bandt & Pompe, 2002 | Detect rhythmicity: FFT dominant frequency, peak amplitude | Identifies tremor, periodic/oscillatory patterns | Noise, spectral leakage |
| Non-linear/complexity features | hfd, antropy (Python packages) | Bandt & Pompe, 2002 | Assess irregularity: Higuchi fractal dimension, permutation entropy | Quantifies complexity, disorder | Computational cost, parameter selection |
| Data merging / label integration | pandas | McKinney, 2010 | Merge clinical labels and extracted features | Enables supervised learning, outcome assignment | Label errors, synchronization issues |
| Imbalance handling | imblearn (SMOTE), sklearn | Chawla et al., 2002 | Address class imbalance (undersampling, SMOTE) | Improves minority class detection | Risk of overfitting, synthetic artifacts |
| Preprocessing (scaling, imputation) | scikit-learn | Pedregosa et al., 2011 / Vasques, 2024 | Normalize, standardize features, impute missing values | Consistent feature scaling, reduces bias | Scaling choice affects outcome |
| Hyperparameter optimization (Grid Search) | scikit-learn | Bergstra & Bengio, 2012 / Pedregosa et al., 2011 / Vasques, 2024 | Systematically search for best hyperparameter combinations | Maximizes predictive performance, reproducibility | Computational cost, risk of overfitting |
| Downstream analysis/classification | scikit-learn, XGBoost, LightGBM | Pedregosa et al., 2011 / Chen & Guestrin, 2016 / Breiman, 2001 / Vasques, 2024 | ML on extracted features: SVM, RF, MLP, KNN, XGB, LGBM, LR | Objective, reproducible symptom classification | Model generalization, overfitting |
| Cross-validation | scikit-learn, iterstrat | Varoquaux et al., 2017 / Sechidis et al., 2011 | Stratified (grouped/multilabel) splits for robust validation | Prevents data leakage, ensures patient independence | Small fold size, variance |
| Performance metrics/evaluation | scikit-learn, numpy | Niculescu-Mizil & Caruana, 2005 | Assess classifiers: F1, ROC-AUC, accuracy, bootstrapped CI | Quantifies model performance, reliability | Metric choice, statistical power |

## Video Processing and Feature Extraction Pipeline

**Video decoding and temporal indexing**

Let a clinical recording be a discrete video sequence $V=\{I_{t}{\}}_{t=1}^{T}$, where $I_{t}\in\mathbb{R}^{H\times W\times3}$is the RGB frame at index $t$, with frame height $H$, width $W$, and $T$total frames. Frames are decoded sequentially using OpenCV. The acquisition frame rate is read from the container metadata ($\mathrm{FPS}$), and each frame is assigned a timestamp

$$\tau_{t}=\frac{\text{CAP\_PROP\_POS\_MSEC}(t)}{1000}\text{ (seconds)},$$

providing the temporal alignment required for subsequent windowing and label synchronization.

**2D pose estimation with YOLOv8x-pose-p6**

For each frame $I_{t}$, we apply a pretrained YOLOv8 pose-estimation model (weights: yolov8x-pose-p6.pt) to infer a 2D human skeleton in a single forward pass. YOLOv8-Pose is a one-stage detector that outputs person detections and associated keypoints. In the COCO-keypoint convention, the model predicts $J=17$anatomical landmarks spanning face, upper limbs, trunk, and lower limbs (nose; left/right eye; left/right ear; left/right shoulder; left/right elbow; left/right wrist; left/right hip; left/right knee; left/right ankle).

Formally, for each detected subject $k$in frame $t$, the model outputs a set of normalized keypoints

$${\hat{\mathbf{p}}}_{t,j}^{\left( k \right)}=(\hat{x}_{t,j}^{\left( k \right)},\hat{y}_{t,j}^{\left( k \right)}),j\in\{1,\ldots,J\},$$

where $\hat{x},\hat{y}\in[0,1]$are expressed in image-normalized coordinates. In our implementation, we retain the first detected person in each frame (i.e., $k=1$) to build a single, consistent pose time series per video.

Computation is executed on GPU (Google Colab).

**Coordinate mapping to pixel space and structured storage**

Normalized coordinates are converted to absolute pixel coordinates by scaling with the original frame dimensions:

$$x_{t,j}=\left\lfloor\hat{x}_{t,j}\text{ }W \right\rfloor,y_{t,j}=\left\lfloor\hat{y}_{t,j}\text{ }H \right\rfloor.$$

This preserves the native image scale and avoids distortions introduced by downstream resampling.

For each frame $t$, we store $\tau_{t}$and the $2J$ pixel coordinates $\left\{ x_{t,j} , y_{t,j}{\}}_{j=1}^{J} \right.$ in a tabular structure (pandas DataFrame), yielding a per-video matrix of size $T\times(1+2J)$. If no pose is detected in a frame (or if keypoint extraction fails), the corresponding keypoint entries are set to missing values (NaN) rather than imputed at this stage; downstream steps handle missingness consistently within the ML pipeline (e.g., imputation inside cross-validation).

**Per-frame displacement signals (radial image-plane magnitude)**

In addition to raw keypoint coordinates, we compute a scalar displacement magnitude for each landmark at each frame. Using the image origin (0,0) as a fixed reference, the displacement signal for landmark $j$ is

$$d_{t,j}=\sqrt{x_{t,j}^{2}+y_{t,j}^{2}}.$$

This produces $J=17$ landmark-specific displacement time series $\left\{ d_{t,j}{\}}_{t=1}^{T} \right.$, which serve as low-level motion signals for later windowed descriptor extraction. This representation is intentionally simple and robust: while it does not represent 3D biomechanics, it quantifies visible movement amplitude in the camera plane and preserves clinically meaningful segmental topography (distal vs proximal; cranial vs appendicular).

**Quality-control artifact: annotated video export**

For traceability and clinical QA, an annotated video is generated by overlaying the predicted skeleton on each frame and writing the resulting frames to an output video file. This provides a human-interpretable verification layer to detect failure modes (e.g., mis-detections, identity switches, occlusion-related dropouts) before feature engineering and model training.

**Output of this stage**

The output of the YOLO stage is therefore a temporally indexed pose time series per video:

1. timestamps $\tau_{t}$;
2. pixel-space keypoint trajectories $\left\{ x_{t,j} , y_{t,j} \right\}$;
3. optional per-keypoint displacement magnitudes $\left\{ d_{t,j} \right\}$;
4. an annotated video for visual QC.

These pose-derived signals and metadata constitute the input to the subsequent stages (windowing, derived descriptor computation, and machine-learning modeling).

## Derived Additional Time-Series Features from landmark trajectories

### Windowed landmark signals

For each video, YOLOv8-Pose provides 2D keypoint trajectories. From these trajectories, the pipeline constructs **per-landmark scalar displacement signals** (one signal per anatomical landmark) that quantify visible motion in the image plane and preserve segmental topography (e.g., distal vs proximal segments). Let $s_{j}(t)$ denote the displacement time series for landmark $j\in\{1,\ldots,J\}$ at frame index $t$. Windows are defined by the expert annotation bounds (« From », « To ») in the exported spreadsheets and correspond to fixed-length segments (10 s in the main protocol). Within each window $w$, we therefore observe a sampled signal In the present analysis, two independent expert raters provided window-level labels (presence = 1 / absence = 0 / uncertain = 2 / mixed within the window) for each of the eight hyperkinetic phenotypes; the consensus label (prim_label), obtained after structured discussion of any discrepancies, served as the operational ground truth, and windows for which the consensus label was uncertain or mixed were excluded before model fitting. Per-phenotype window counts (annotated, positive, negative, uncertain, mixed) are reported in Supplementary Table S2 of the main manuscript, and Cohen's κ inter-rater agreement at the window level prior to consensus is reported per phenotype in Supplementary Table S3.

$$\mathbf{s}_{j}^{\left( w \right)}=\{s_{j}(t){\}}_{t=1}^{N_{w}},$$

with $N_{w}$frames and sampling frequency $f_{s}$ (FPS; set to 30 Hz in the implementation). The feature extraction procedure operates independently on each window and each landmark signal.

**Discrete derivatives and event-sensitive transforms**

To capture temporal dynamics beyond amplitude, the method computes finite-difference derivatives within each window:

**First difference (velocity proxy).** With $s(t)$ denoting one landmark displacement signal within a window, the discrete first difference used in the code is

$$\Delta s(t)=s(t)-s(t-1),t=2,\ldots,N,$$

with $\Delta s(1)=0$ implemented via a prepend convention.

**Second difference magnitude (acceleration proxy).** The “acceleration” surrogate is defined as the absolute second finite difference:

$$a(t)=\mid\Delta s(t)-\Delta s(t-1)\mid,t=2,\ldots,N,$$

again with a prepend convention for the initial value. This quantity is particularly sensitive to abrupt changes and jerk-like events.

**Feature families and mathematical definitions (per landmark, per window)**

For each landmark displacement signal $s(t)$ in a given window, a feature vector is computed comprising **distributional**, **temporal/kinematic**, **spectral**, and **nonlinear complexity** descriptors, plus short-horizon **rolling-mean** summaries. All features are computed directly from the windowed sample (no padding), and signals are cast to floating point; windows with fewer than two samples are skipped.

**Distributional amplitude and shape descriptors**

These quantify the “movement burden” and variability in a window.

- **Mean:** $\mu=\frac{1}{N}\sum_{t=1}^{N} s(t)$
- **Standard deviation:** $\sigma=\sqrt{\frac{1}{N}\sum_{t=1}^{N} (s(t)-\mu)^{2}}$
- **Variance:** $\mathrm{Var}(s)=\sigma^{2}$
- **Median:** $\mathrm{median}(s)$
- **Minimum / Maximum:** $\min_{t}s(t)$, $\max_{t}s(t)$
- **Range (peak-to-peak):** $\max_{t}s(t)-\min_{t}s(t)$
- **Interquartile range (IQR):** $Q_{0.75}(s)-Q_{0.25}(s)$
- **Energy:** $E=\sum_{t=1}^{N} s(t)^{2}$

Additionally, two standardized shape moments are computed:

- **Skewness:** $\gamma_{1}=\mathbb{E}\text{ }\left[ \left( \frac{s-\mu}{\sigma} \right)^{3} \right]$(sample estimate)
- **Excess kurtosis:** $\gamma_{2}=\mathbb{E}\text{ }\left[ \left( \frac{s-\mu}{\sigma} \right)^{4} \right]-3$(sample estimate)

These descriptors summarize baseline level, dispersion, and tail behavior (e.g., rare high-amplitude excursions).

**Temporal evolution and direction-change proxies**

These quantify slow drift and rapid alternations.

- **Linear trend (slope):** the slope of the least-squares fit

$$s(t)\approx\beta_{0}+\beta_{1}t,\text{feature }=\beta_{1}.$$

- **Direction-change propensity (zero crossings of the first difference):** the number of sign reversals in $\Delta s(t)$, computed as

$$Z=\sum_{t=2}^{N-1} \mathbf{1}\{\Delta s(t)\cdot\Delta s(t+1)<0\}.$$

This counts repeated alternations of movement direction in the displacement trajectory.

- **Mean absolute “acceleration”:**

$$a=\frac{1}{N}\sum_{t=1}^{N} a(t),$$

where $a(t)=\mid\Delta s(t)-\Delta s(t-1)\mid$. This increases with abrupt changes and paroxysmal events.

combined_hmd

**Spectral rhythmicity descriptors (FFT-based)**

To quantify oscillatory structure, the pipeline computes the discrete Fourier transform of the windowed signal:

$$S(k)=\sum_{t=0}^{N-1} s(t)\text{ }e^{-i2\pi kt/N},k=0,\ldots,N-1.$$

Let $A(k)=\mid S(k)\mid$be the magnitude spectrum, and let the corresponding frequency grid be

$$f(k)=\frac{k}{N}\text{ }f_{s}\text{(with negative-frequency wrap as in fftfreq}).$$

The method excludes the DC component $k=0$ and finds the dominant oscillatory component via

$$k^{\text{\textbackslash*}}=\arg\max_{k\in\{1,\ldots,N-1\}}A(k).$$

Two spectral features are retained:

- **Peak frequency:** $f_{\text{peak}}=f(k^{\text{\textbackslash*}})$
- **Peak amplitude:** $A_{\text{peak}}=A(k^{\text{\textbackslash*}})$

These are intended to capture rhythmic movement (e.g., tremor-like periodicity) while remaining lightweight and interpretable in short windows.

**Entropy of amplitude distribution (histogram-based)**

A Shannon entropy is computed from a fixed-bin histogram of the windowed samples. Let $\left\{ b_{m}{\}}_{m=1}^{10} \right.$ be 10 histogram bins over the observed range of $s(t)$, with density normalization. The method constructs a discrete distribution $p_{m}$ (with a small $\varepsilon$added for numerical stability), then computes

$$H=-\sum_{m=1}^{10} p_{m}\log(p_{m}+\varepsilon).$$

This increases when the amplitude distribution is broader and less concentrated around a set-point.

**Nonlinear irregularity / complexity descriptors**

Two complementary measures are computed to characterize higher-order irregularity beyond variance or periodicity.

**Higuchi fractal dimension (HFD).** For each $k\in\{1,\ldots,k_{\max}\}$(with $k_{\max}=5$), the algorithm forms $k$ sub-series indexed by $m\in\{0,\ldots,k-1\}$ and computes a length estimate $L_{m}(k)$ based on averaged absolute increments sampled every $k$ points, with the normalization used in the implementation. The average length $L(k)=\frac{1}{k}\sum_{m} L_{m}(k)$ is then regressed in log–log space:

$$\log L(k)\approx c-D\log k,$$

and the feature returned is $D$ (implemented as the negative slope of $\log L(k)$vs $\log k$). Larger values indicate more scale-invariant irregularity in the trajectory.

**Permutation entropy (PE).** With embedding order $m=3$ and delay $\tau=1$, the method converts local length-$m$ segments $\left( s(t),s(t+\tau),s(t+2\tau) \right)$ into ordinal patterns (permutations) according to their rank order. Let $p(\pi)$ be the empirical probability of pattern $\pi$over the window. Permutation entropy is

$$\mathrm{PE}=-\sum_{\pi} p(\pi)\log(p(\pi)+\varepsilon).$$

Higher PE corresponds to less predictable local ordering and greater temporal disorder.

**Short-horizon rolling-mean summaries (sustained bias)**

To separate sustained deviations from transient spikes, the pipeline computes centered rolling means with window sizes $w\in\{3,5,7\}$ frames:

$$r_{w}(t)=\frac{1}{\mid\mathcal{N}_{w}(t)\mid}\sum_{u\in\mathcal{N}_{w}(t)} s(u),$$

where $\mathcal{N}_{w}(t)$is the centered neighborhood of width $w$(with edge handling via $\text{min\_periods}=1$). The exported feature is the average of $r_{w}(t)$over time:

$$r_{w}=\frac{1}{N}\sum_{t=1}^{N} r_{w}(t).$$

These descriptors emphasize persistent displacement within the window while reducing sensitivity to isolated artefacts.

**Feature naming and dimensionality**

Features are stored using a transparent naming convention combining the landmark displacement channel name (e.g., right_wrist_distance) and the descriptor suffix (e.g., _fft_peak_freq, _higuchi_fd). For each window and each landmark displacement signal, this implementation computes **19 primary descriptors** (distributional + temporal + spectral + entropy + complexity) and **3 rolling-mean descriptors**, yielding **22 features per landmark per window**. With $J=17$ landmarks, this corresponds to $22\times17=374$ derived features per window in the multi-label pipeline.

## Window-Based Binary Classification of Individual HMD Presence Versus Absence

This section describes the **binary, window-level screening pipeline** used to detect the presence of a single HMD phenotype in **10-second segments**, as implemented in windows.py.

**Window definition, quality filtering, and label construction**

For each recording (one subject per file), the pose-derived time series are already organized in a tabular structure containing (i) **keypoint displacement channels** (17 anatomical landmarks) and (ii) clinical annotation columns per phenotype. A **window** is defined by the pair $\left( \mathrm{From} , \mathrm{To} \right)$and corresponds to a contiguous time interval of fixed duration (10 s in the study protocol). All samples belonging to the same $\left( \mathrm{From} , \mathrm{To} \right)$pair form one group $w$.

Let $a_{t}^{\left( \mathcal{l} \right)}\in\{0,1,2\}$ denote the rater-provided frame-level annotation for phenotype $\mathcal{l}$at time index $t$within window $w$. The script enforces two key filtering rules before feature extraction:

1. **Uncertainty exclusion**: any window containing at least one uncertain label ($a_{t}^{\left( \mathcal{l} \right)}=2$) is removed:

$$w\text{ is valid }\Longleftrightarrow\text{ }\forall t\in w,\text{ }a_{t}^{\left( \mathcal{l} \right)}\neq2.$$

1. **Completeness of pose-derived channels**: any window containing missing values in the displacement channels is removed.

After filtering, a **binary window label** is assigned using an “any-positive” rule:

$$y_{w}^{\left( \mathcal{l} \right)}=\mathbb{1}\left[ \exists\text{ }t\in w\text{ such that }a_{t}^{\left( \mathcal{l} \right)}=1 \right].$$

This yields $y_{w}^{\left( \mathcal{l} \right)}\in\{0,1\}$per window $w$and phenotype $\mathcal{l}$.

**Screening-oriented negative class (controls only)**

The pipeline is explicitly configured as a **screening-style case–control contrast**: when a window is labeled negative ($y_{w}^{\left( \mathcal{l} \right)}=0$) and the recording belongs to a patient (i.e., not a control subject), that window is **excluded** from the dataset. Only **control** windows contribute to the negative class. Formally, if $g(w)$ denotes the subject identity for window $w$, and $\mathcal{C}$is the set of control subjects, then:

$$\text{keep }w\text{ }\Longleftrightarrow\text{ }\left( y_{w}^{\left( \mathcal{l} \right)} = 1 \right)\text{ }\vee\text{ }\left( g(w)\in\mathcal{C} \right).$$

This design prevents “symptom-absent patient windows” from being used as negatives and forces the classifier to learn a discrimination between **symptom-expressing patient windows** (positives) and **healthy control windows** (negatives).

**Feature representation per window**

For each valid window $w$, the script extracts engineered descriptors from the 17 displacement channels (one per landmark), producing a fixed-dimensional vector $\mathbf{x}_{w}\in\mathbb{R}^{d}$. The feature extractor includes distributional statistics (mean, std, range, skewness, kurtosis, energy, IQR), temporal proxies (slope, zero-crossings, mean absolute second difference), spectral features (FFT peak frequency/amplitude), and nonlinear complexity metrics (Higuchi fractal dimension and permutation entropy), along with short-horizon rolling mean summaries.

The resulting dataset for phenotype $\mathcal{l}$ is:

$$\mathcal{D}^{\left( \mathcal{l} \right)}=\left\{ \left( \mathbf{x}_{w},\text{ }y_{w}^{\left( \mathcal{l} \right)},\text{ }g(w) \right) \right\}_{w=1}^{N},$$

where $g(w)$is the subject identifier (group label) used for leakage-free splitting.

**Leakage-free cross-validation at the subject level**

To prevent leakage between training and testing, the evaluation uses **StratifiedGroupKFold** (5 folds) with shuffling and a fixed random seed. The split produces indices $\mathcal{I}_{\text{train}}^{\left( k \right)}$, $\mathcal{I}_{\text{test}}^{\left( k \right)}$such that:

$$g(w)\in\text{train fold }\Rightarrow\text{ }g(w)\notin\text{test fold}$$

for every subject, while attempting to preserve the class ratio in each fold (stratification on $y$).

**Training-time class balancing (within each fold)**

Because the screening dataset may be imbalanced, the script applies a **fold-local undersampling heuristic** on the training windows. Let $n_{0}$and $n_{1}$be the counts of negative and positive training windows, respectively. If the relative imbalance exceeds 20%:

$$\frac{\mid n_{0}-n_{1}\mid}{\max(n_{0},n_{1})}>0.2,$$

the majority class is randomly resampled down to match the minority class count:

$$\mathcal{D}_{\text{train}}^{\text{bal}}=\text{Resample}\left( \mathcal{D}_{\text{train}} \right),n_{0}\approx n_{1}.$$

This balancing is applied **only on the training partition** of each fold and never on the test set.

**Preprocessing (scaling) and numeric stability**

For each fold, a feature scaler $S(\cdot)$is fit on the balanced training features and applied to both train and test features:

$$\tilde{\mathbf{x}}=S(\mathbf{x}).$$

The pipeline benchmarks several scalers (StandardScaler, MinMaxScaler, RobustScaler, PowerTransformer). After scaling, numerical stability is enforced by mapping NaNs and infinities to finite values ($0$ for NaN and bounded large magnitudes for $\pm\infty$).

**Model families, hyperparameter enumeration, and probability calibration**

For each phenotype $\mathcal{l}$, we benchmarked a set of representative classifier families. Each model defines a scoring function $f_{\theta}:\mathbb{R}^{d}\to\mathbb{R}$ (or directly a probability) applied to the window feature vector $\mathbf{x}_{w}$. When a calibrated probability was available, we denote

$$\hat{p}_{w}=\Pr_{\theta}(y_{w}^{\left( \mathcal{l} \right)}=1\mid\mathbf{x}_{w})\in[0,1],$$

and use $\hat{p}_{w}$for ROC-AUC computation and thresholded predictions for F1/accuracy. All hyperparameters were explored via an explicit Cartesian product enumeration (scikit-learn ParameterGrid) and evaluated under subject-grouped cross-validation; performance was summarized by the mean of fold-wise metrics.

#### Gradient-boosted decision trees (XGBoost)

We evaluated XGBoost gradient boosting over trees, where the model is an additive ensemble

$$f_{\theta}(\mathbf{x})=\sum_{m=1}^{M} \eta\text{ }h_{m}(\mathbf{x}),$$

with $h_{m}$ decision trees and learning rate $\eta$. The tested hyperparameter grid was: number of trees $M\in\{100,200,300\}$, maximum depth $\in\{6\}$, learning rate $\in\{0.05,0.1\}$, subsampling ratio $\in\{1.0\}$, column subsampling $\in\{1.0\}$, minimum child weight $\in\{1,5\}$, and split loss regularization $\gamma\in\{0,1\}$.

#### Gradient-boosted decision trees (LightGBM)

We evaluated LightGBM, a histogram-based gradient boosting implementation. The tested grid was: number of trees $\in\{100,200,300\}$, number of leaves $\in\{31,63\}$, learning rate $\in\{0.05,0.1\}$, max depth $\in\{-1\}$(unconstrained), feature fraction $\in\{1.0\}$, bagging fraction $\in\{1.0\}$, minimum child samples $\in\{10\}$, and $\mathcal{l}_{2}$ regularization $\lambda\in\{0.1,1.0\}$.

#### Random Forests

We evaluated RandomForest classifiers, i.e., bagged ensembles of decision trees:

$$\hat{p}(\mathbf{x})=\frac{1}{M}\sum_{m=1}^{M} \hat{p}_{m}(\mathbf{x}),$$

where $\hat{p}_{m}$is the class probability from tree $m$. The tested grid was: number of trees $M\in\{100,200,300\}$, maximum depth $\in\{\text{None}\}$(unconstrained), minimum samples to split $\in\{2\}$, minimum samples per leaf $\in\{1\}$, and feature subsampling rule $\in\{\text{sqrt}\}$.

#### Support Vector Machine (RBF kernel)

We evaluated SVMs with a radial basis function kernel:

$$K(\mathbf{x},\mathbf{x}^{'})=\exp\text{ ⁣}\left( -\gamma\parallel\mathbf{x}-\mathbf{x}^{'}\parallel^{2} \right),$$

with penalty parameter $C$. The grid was: $C\in\{1,10\}$, kernel fixed to RBF, and $\gamma\in\{\text{scale},0.01\}$. Probabilistic outputs were enabled (probability=True) and further probability calibration was applied as described below.

#### Logistic Regression

We evaluated $\mathcal{l}_{2}$-regularized logistic regression, where

$$\hat{p}(\mathbf{x})=\sigma(\mathbf{w}^{\top}\mathbf{x}+b),\sigma(z)=\frac{1}{1+e^{-z}}.$$

The tested grid was: inverse regularization strength $C\in\{1\}$, penalty $\in\{\mathcal{l}_{2}\}$, solver $\in\{\text{lbfgs}\}$, and maximum iterations $\in\{1000\}$.

#### k-Nearest Neighbors (KNN)

We evaluated KNN with Euclidean distance ($p=2$). For a query $\mathbf{x}$, predictions are derived from the label distribution among the $k$nearest training points. The grid was: $k\in\{5,7\}$, weights $\in\{\text{uniform}\}$, Minkowski power $p\in\{2\}$.

#### Multi-Layer Perceptron (MLP)

We evaluated a feedforward neural network classifier with ReLU nonlinearity. The grid was: hidden layer sizes $\in\{(128,),(64,64)\}$, weight decay parameter $\alpha\in\{0.01,0.0001\}$, activation $\in\{\text{relu}\}$, solver $\in\{\text{adam}\}$, initial learning rate $\in\{0.001\}$, and batch size $\in\{64\}$.

**Probability calibration (applied to SVM and Random Forest variants)**

To ensure well-behaved probabilistic outputs for ROC-AUC and downstream analyses, models in the SVM and RandomForest families were additionally wrapped in sigmoid calibration (CalibratedClassifierCV, 3-fold internal calibration). Concretely, for a base model score $f(\mathbf{x})$, calibrated probabilities are obtained via Platt scaling:

$$\hat{p}(\mathbf{x})=\sigma\text{ }\left( a\text{ }f(\mathbf{x})+b \right),$$

where $a,b$are learned on calibration folds. Calibration is performed strictly within each outer training fold to avoid test leakage.

**Window-level evaluation metrics**

Within each fold, the trained model produces:

- predicted labels $\hat{y}_{w}\in\{0,1\}$,
- predicted probabilities $\hat{p}_{w}=\Pr(\hat{y}_{w}=1)$.

The script reports per-fold:

- **F1-score** for class 1 and class 0 (from classification_report),

$$F1=\frac{2\cdot\mathrm{Precision}\cdot\mathrm{Recall}}{\mathrm{Precision}+\mathrm{Recall}},$$

- **Accuracy**,

$$\mathrm{Acc}=\frac{1}{N}\sum_{w} \mathbb{1}[\hat{y}_{w}=y_{w}],$$

- **ROC-AUC** computed from $\hat{p}_{w}$.

Fold-level metrics are averaged across valid folds (folds lacking both classes are skipped to avoid degenerate AUC estimation).

**Optional subject-level voting (derived from window predictions)**

Although the primary task is window-level screening, the script also derives a **subject-level decision** by aggregating window predictions for each subject in the test folds.

For a subject $g$, let $\left\{ \hat{y}_{w}{\}}_{w\in\mathcal{W}(g)} \right.$be the set of predicted window labels for that subject across test windows. The subject-level predicted label is computed by majority vote (equivalently, mean threshold at 0.5):

$$\hat{Y}_{g}=\mathbb{1}\left[ \frac{1}{\mid\mathcal{W(}g)\mid}\sum_{w\in\mathcal{W(}g)} \hat{y}_{w} \geq0.5 \right].$$

The subject-level “ground truth” is defined as:

$$Y_{g}=\mathbb{1}\left[ \exists\text{ }w\in\mathcal{W}(g)\text{ with }y_{w}=1 \right],$$

i.e., a subject is positive if any of their retained windows is positive. Subject-level accuracy, sensitivity, and specificity are then computed from $\left( \hat{Y}_{g} , Y_{g} \right)$across evaluated subjects.

## Multi-label detection of concurrent HMD phenotypes

### Problem formulation and data representation

Let $L$denote the number of HMD phenotypes (here $L=8$). Each clinical video is partitioned into non-overlapping (annotation-defined) 10-s windows $w$, and each window is represented by a feature vector $\mathbf{x}_{w}\in\mathbb{R}^{d}$computed from pose-derived kinematic descriptors. Each window carries a multi-label target vector

$$\mathbf{y}_{w}=(y_{w}^{\left( 1 \right)},\ldots,y_{w}^{\left( L \right)})\in\{0,1{\}}^{L},$$

where $y_{w}^{\left( \mathcal{l} \right)}=1$ indicates presence of phenotype $\mathcal{l}$in that window and $0$indicates absence. Windows with uncertain clinical annotation are excluded to preserve a strictly binary ground truth for supervised learning.

To reduce rater idiosyncrasies for the multi-label task, we conduct all multi-label analyses on a consensus reference dataset obtained by harmonizing the independent clinician ratings into a single window-level multi-label ground truth.

We retain control subjects and (optionally) symptom-absent windows (“all-zero” label vectors) to anchor the negative class with normative movement, which is critical for control-aware operating regimes and calibration of false-alarm constraints.

**Patient-level grouping and leakage-free cross-validation**

Windows are not independent because many windows belong to the same subject. We therefore enforce group-wise splitting by subject (patient/control identifier) to prevent train–test leakage.

Define a subject index mapping $g(w)\in\{1,\ldots,N\}$ indicating the subject associated with window $w$. For subject $s$, define its aggregated (OR) label vector used for stratification:

$$\mathbf{Y}_{s}=\max_{w:g(w)=s}\mathbf{y}_{w}\text{(element-wise OR across windows).}$$

Cross-validation partitions are generated at the subject level using multilabel-stratified K-fold splitting (preserving the joint prevalence structure across labels as much as possible), then expanded back to the corresponding window sets for model training and evaluation.

This ensures that no subject contributes windows to both training and test folds, and that prevalence of rare phenotypes is controlled at the subject level rather than at the window level (which would otherwise inflate effective sample size and bias estimates).

**Multi-label learning strategy: binary relevance with calibrated probabilities**

We implement multi-label prediction via **binary relevance**: one probabilistic binary classifier per label $\mathcal{l}$, trained on all windows with that label’s binary targets. Concretely, for each $\mathcal{l}\in\{1,\ldots,L\}$ we learn a scoring function $f_{\mathcal{l}}(\mathbf{x})$ and an associated probability model

$$\hat{p}_{w}^{\left( \mathcal{l} \right)}=\Pr(y_{w}^{\left( \mathcal{l} \right)}=1\mid\mathbf{x}_{w})\in[0,1].$$

The window-level probability vector is ${\hat{\mathbf{p}}}_{w}=(\hat{p}_{w}^{\left( 1 \right)},\ldots,\hat{p}_{w}^{\left( L \right)})$.

Because several strong baselines (e.g., linear SVM variants) do not natively output calibrated probabilities, we apply explicit probability extraction rules: (i) use predict_proba when available; (ii) otherwise use calibrated decision scores via sigmoid calibration (CalibratedClassifierCV) when feasible; and (iii) fall back to a logistic mapping of the decision function,

$$\hat{p}=\sigma(z)=\frac{1}{1+e^{-z}},$$

when calibration is not statistically feasible for rare labels (e.g., too few positives for K-fold calibration).

**Per-label preprocessing, feature selection, and imbalance handling (within fold)**

Within each outer training fold, each label-specific pipeline can include:

1. **Scaling/normalization** (label-agnostic transform but selected per candidate pipeline).
2. **Univariate feature selection** via mutual information: Select the top $k$features maximizing $I(X_{j};y^{\left( \mathcal{l} \right)})$, yielding a reduced vector ${\tilde{\mathbf{x}}}_{w}\in\mathbb{R}^{k_{\mathcal{l}}}$. Label-specific $k_{\mathcal{l}}$is allowed to reflect phenotype-dependent signal sparsity.
3. **Class rebalancing** through (optional) SMOTE variants restricted to the training fold only, with safeguards that adapt $k$-neighbors to the minority count and avoid synthetic sampling when infeasible.
4. **Class weighting** (e.g., balanced weights) to reduce bias toward the majority class when prevalence is low.

All of these operations are performed inside the training data of each fold to avoid leakage of distributional information from the test subjects.

**From window probabilities to patient-level multi-label profiles**

Clinical interpretation and downstream reporting are performed at the **patient level**, not at the window level, because clinical phenotyping is ultimately a subject-level decision (presence/absence of each phenotype for a patient). We therefore aggregate repeated short observations (10-s windows) into a patient-level probability vector.

For subject $s$, let $W_{s}=\{w:g(w)=s\}$ denote its set of windows, with cardinality $\mid W_{s}\mid=n_{s}$. For each label $\mathcal{l}$, we aggregate the set $\left\{ \hat{p}_{w}^{\left( \mathcal{l} \right)}:w\in W_{s} \right\}$ into a patient-level probability $\hat{\pi}_{s}^{\left( \mathcal{l} \right)}$using one of the following operators:

**Percentile pooling (p90; primary).**

$$\hat{\pi}_{s}^{\left( \mathcal{l} \right)}=\mathrm{Quantile}_{q}(\{\hat{p}_{w}^{\left( \mathcal{l} \right)}{\}}_{w\in W_{s}}),q=0.90.$$

This implements a robust “high-evidence” summary: the patient-level score reflects the typical upper tail of window evidence rather than the single maximum (which can be unstable) or the mean (which can dilute intermittent phenomena).

For completeness, the pipeline also supports:

**Max pooling:** $\hat{\pi}_{s}^{\left( \mathcal{l} \right)}=\max_{w\in W_{s}}\hat{p}_{w}^{\left( \mathcal{l} \right)}$.

**Top-**$k$ **mean pooling:** $\hat{\pi}_{s}^{\left( \mathcal{l} \right)}=\frac{1}{k}\sum_{i\in\text{Top}k} \hat{p}_{w_{i}}^{\left( \mathcal{l} \right)}$, where Top$k$ are the $k$largest window probabilities.

**Noisy-OR pooling (event accumulation):**

$$\hat{\pi}_{s}^{\left( \mathcal{l} \right)}=1-\prod_{w\in W_{s}} (1-\hat{p}_{w}^{\left( \mathcal{l} \right)}),$$

which approximates the probability that at least one window contains the phenotype if window events are treated as conditionally independent given the model.

Ground-truth patient labels for evaluation are defined consistently as:

$$Y_{s}^{\left( \mathcal{l} \right)}=\max_{w\in W_{s}}y_{w}^{\left( \mathcal{l} \right)}.$$

### Label-wise thresholding tuned on training subjects under control-aware constraints

Patient-level probabilities ${\hat{\boldsymbol{\pi}}}_{s}$are converted into binary patient-level calls via **label-specific thresholds** $\tau_{\mathcal{l}}$, chosen exclusively on the training subjects of each fold:

$$\hat{Y}_{s}^{\left( \mathcal{l} \right)}=\mathbb{I}[\hat{\pi}_{s}^{\left( \mathcal{l} \right)}\geq\tau_{\mathcal{l}}].$$

Thresholds are optimized over a dense grid $\mathcal{T}\subset(0,1)$(e.g., 199 values between 0.01 and 0.99).

Crucially, threshold selection is **control-aware**. Let $\mathcal{S}_{\text{ctrl}}$be the subset of control subjects in the training fold (identified by subject IDs), and $\mathcal{S}_{\text{all}}$all training subjects. For a candidate threshold $t$, define confusion counts on the training set:

$$\text{TN}(t),\text{FP}(t),\text{FN}(t),\text{TP}(t),$$

and corresponding rates:

$$\text{FPR}(t)=\frac{\text{FP}(t)}{\text{FP}(t)+\text{TN}(t)},\text{FNR}(t)=\frac{\text{FN}(t)}{\text{FN}(t)+\text{TP}(t)},\text{Spec}(t)=1-\text{FPR}(t),\text{Rec}(t)=1-\text{FNR}(t).$$

Additionally, compute **control-only** false positives $\text{FP}_{\text{ctrl}}(t)$and $\text{FPR}_{\text{ctrl}}(t)$restricted to $\mathcal{S}_{\text{ctrl}}$.

We support several threshold policies; the primary one is a **clinical-cost objective** with optional label-specific weighting:

$$\mathcal{C}_{\mathcal{l}}(t)=(1-\alpha_{\mathcal{l}})\text{ }\text{FPR}_{\mathcal{l}}(t)+\alpha_{\mathcal{l}}\text{ }\text{FNR}_{\mathcal{l}}(t),$$

and we select

$$\tau_{\mathcal{l}}=\arg\min_{t\in\mathcal{T}\cap\text{Feasible}_{\mathcal{l}}}\mathcal{C}_{\mathcal{l}}(t),$$

where $\alpha_{\mathcal{l}}\in[0,1]$prioritizes false negatives ($\alpha_{\mathcal{l}}\uparrow$) versus false positives ($\alpha_{\mathcal{l}}\downarrow$). Feasibility enforces clinically motivated constraints, including: (i) maximum allowed number of control false positives, (ii) maximum control FPR, and/or (iii) minimum specificity targets, with label-specific targets permitted for rare/high-impact phenotypes.

This design yields phenotype-specific operating points aligned with clinical priorities (e.g., conservative behavior for rare labels, stricter control false-alarm limits for screening deployment).

**Model selection: global comparators and per-label error-minimizing selection (optional nested layer)**

Two complementary selection paradigms are used:

1. **Global pipeline selection** (single configuration across labels) based on a comparator metric (macro-AUPRC, macro-AUC, sample-wise Jaccard, Hamming accuracy, or a clinical-cost summary), computed at the patient level and averaged across folds.
2. **Per-label “best model” selection** (one pipeline per phenotype) that minimizes patient-level errors for that label. Concretely, for label $\mathcal{l}$ and candidate configuration $c$, compute the patient-level error rate

$$\text{Err}_{\mathcal{l}}^{\left( c \right)}=\frac{\text{FP}_{\mathcal{l}}^{\left( c \right)}+\text{FN}_{\mathcal{l}}^{\left( c \right)}}{\mid\mathcal{S}\mid},$$

under the same aggregation (p90) and thresholding mechanism, and select the configuration achieving minimal $\text{Err}_{\mathcal{l}}^{\left( c \right)}$under the desired constraints.

In the implementation, this can be embedded in an inner (nested) cross-validation loop to prevent optimistic bias when selecting per-label models and thresholds.

**Patient-level multi-label evaluation metrics**

All primary metrics are computed at the **patient level** from $\mathbf{Y}_{s}$and ${\hat{\mathbf{Y}}}_{s}$, with discrimination metrics computed from ${\hat{\boldsymbol{\pi}}}_{s}$:

- **Macro ROC-AUC** and **macro AUPRC** computed label-wise and averaged across labels, with “safe” handling that skips labels lacking both classes in a fold.
- **Micro-F1** and **macro-F1** on thresholded outputs.
- **Hamming loss**:

$$\text{HL}=\frac{1}{NL}\sum_{s=1}^{N} \sum_{\mathcal{l}=1}^{L} \mathbb{I}[\hat{Y}_{s}^{\left( \mathcal{l} \right)}\neq Y_{s}^{\left( \mathcal{l} \right)}],\text{Hamming accuracy}=1-\text{HL}.$$

- **Sample-wise Jaccard index** averaged over subjects:

$$J=\frac{1}{N}\sum_{s=1}^{N} \frac{\mid{\hat{\mathcal{L}}}_{s}\cap\mathcal{L}_{s}\mid}{\mid{\hat{\mathcal{L}}}_{s}\cup\mathcal{L}_{s}\mid},$$

where $\mathcal{L}_{s}=\{\mathcal{l}:Y_{s}^{\left( \mathcal{l} \right)}=1\}$and ${\hat{\mathcal{L}}}_{s}=\{\mathcal{l}:\hat{Y}_{s}^{\left( \mathcal{l} \right)}=1\}$.

- **Exact match ratio**: fraction of subjects with perfectly correct multi-label profiles ${\hat{\mathbf{Y}}}_{s}=\mathbf{Y}_{s}$.

Finally, because clinical deployment requires explicit false-alarm accounting in healthy individuals, we report **control-only** false positives and control FPR per label on held-out folds, in addition to overall specificity.

## Features Importance

This section describes the feature-importance procedure implemented in features.py. The goal is to quantify, for each phenotype, **which engineered kinematic descriptors most strongly influence the final patient-level decision**, under the exact same inference mechanism used in the main multi-label pipeline (window probabilities $\to$patient aggregation $\to$label-wise thresholding).

**What “importance” measures in our setting**

Our deployed decision rule for a given label $\mathcal{l}$ is evaluated at the **patient level**. For patient $s$, we first compute window-level probabilities $\hat{p}_{w}^{\left( \mathcal{l} \right)}$ for all windows $w\in W_{s}$, then aggregate them using percentile pooling (p90) to obtain a patient probability $\hat{\pi}_{s}^{\left( \mathcal{l} \right)}$, and finally threshold:

$$\hat{\pi}_{s}^{\left( \mathcal{l} \right)}\text{ }=\text{ }Q_{0.90}(\{\hat{p}_{w}^{\left( \mathcal{l} \right)}{\}}_{w\in W_{s}}),\hat{Y}_{s}^{\left( \mathcal{l} \right)}\text{ }=\text{ }\mathbb{1}[\hat{\pi}_{s}^{\left( \mathcal{l} \right)}\geq\tau_{\mathcal{l}}].$$

Feature importance is defined **with respect to the final patient-level classification error** produced by this full chain. Concretely, for each label $\mathcal{l}$ we use the patient-level error rate:

$$\mathrm{Err}^{\left( \mathcal{l} \right)}\text{ }=\text{ }\frac{\mathrm{FP}^{\left( \mathcal{l} \right)}+\mathrm{FN}^{\left( \mathcal{l} \right)}}{N_{\text{patients}}}.$$

This is a direct “how many patients are misclassified” objective, which is aligned with clinical deployment constraints and complements discrimination metrics (AUC/AUPRC).

**Strict leakage avoidance: importance computed on outer test folds only**

Feature importance is computed **only on held-out outer test folds**, never on training data. For each outer fold $k$:

1. Fit the complete label-specific pipeline on the **outer training windows** (including scaler, optional SelectKBest, optional SMOTE, classifier).
2. Tune label-wise patient thresholds $\tau_{\mathcal{l}}$ **using only outer-training patients** (with control-aware constraints as configured).
3. Compute feature importance by permuting test-fold features and measuring the change in **patient-level error** on the same outer test patients.

This design ensures that both the model parameters and the thresholds are fixed **before** importance is measured, and that importance reflects genuine out-of-sample behavior.

**Baseline patient-level predictions on the test fold**

Fix one phenotype $\mathcal{l}$ and one outer fold $k$. Let $X_{\text{test}}\in\mathbb{R}^{n\times d}$be the matrix of test windows (rows are windows; columns are features), $g\in\{1,\ldots,N{\}}^{n}$the corresponding patient IDs, and $y_{\text{test}}^{\left( \mathcal{l} \right)}\in\{0,1{\}}^{n}$ the window labels for $\mathcal{l}$.

**Baseline window probabilities.** We compute per-window probabilities using the fitted pipeline:

- If the pipeline exposes predict_proba, use $\hat{p}^{\left( \mathcal{l} \right)}=\text{predict\_proba}(X_{\text{test}})_{\left[ :,1 \right]}$.
- Else if it exposes decision_function, map scores to $\left[ 0 , 1 \right]$with a sigmoid:

$$\hat{p}^{\left( \mathcal{l} \right)}=\sigma(z)=\frac{1}{1+e^{-z}}.$$

- Else fall back to hard predictions cast to float.

**Patient ground truth.** Patient-level labels are the OR across that patient’s windows:

$$Y_{s}^{\left( \mathcal{l} \right)}=\max_{w\in W_{s}}y_{w}^{\left( \mathcal{l} \right)}.$$

**Patient predictions.** We aggregate the window probabilities to patient-level probabilities via p90, then threshold with the already-trained $\tau_{\mathcal{l}}$from the outer training fold:

$$\hat{Y}_{s}^{\left( \mathcal{l} \right)}=\mathbb{1}\left[ Q_{0.90}(\{\hat{p}_{w}^{\left( \mathcal{l} \right)}{\}}_{w\in W_{s}})\geq\tau_{\mathcal{l}} \right].$$

Finally, compute the baseline patient-level error $\mathrm{Err}_{\text{base}}^{\left( \mathcal{l} \right)}$.

**Permutation importance: definition and algorithm**

For a given feature index $j$(column $j$of $X_{\text{test}}$), permutation importance measures how much performance deteriorates when the relationship between that feature and the outcome is broken.

**Permutation operator.** For one repeat $r$, we create a perturbed test matrix $X_{\text{test}}^{\left( j , r \right)}$by applying a random permutation $\pi$to the rows of feature $j$:

$$X_{\text{test}}^{\left( j , r \right)}[i,j]\text{ }=\text{ }X_{\text{test}}[\pi(i),j],X_{\text{test}}^{\left( j , r \right)}[i,m]\text{ }=\text{ }X_{\text{test}}[i,m]\text{ for }m\neq j.$$

All other features remain unchanged; the marginal distribution of feature $j$is preserved, but its alignment with labels (and its joint structure with other predictors) is disrupted, thereby testing how much the trained model relies on that feature.

**Recompute the full patient-level decision.** Using the same fixed pipeline and the same fixed threshold $\tau_{\mathcal{l}}$, we obtain:

1. window probabilities $\hat{p}_{w,(j,r)}^{\left( \mathcal{l} \right)}$from $X_{\text{test}}^{\left( j , r \right)}$,
2. patient-level probabilities by p90,
3. patient-level predictions $\hat{Y}_{s,(j,r)}^{\left( \mathcal{l} \right)}$,
4. permuted patient error $\mathrm{Err}_{\left( j , r \right)}^{\left( \mathcal{l} \right)}$.

**Importance score.** The importance of feature $j$ for label $\mathcal{l}$ in fold $k$ is defined as the mean increase in patient error rate across repeats:

$$\Delta_{j}^{\left( \mathcal{l} \right)}\text{ }=\text{ }\frac{1}{R}\sum_{r=1}^{R} \left( \mathrm{Err}_{\left( j , r \right)}^{\left( \mathcal{l} \right)}-\mathrm{Err}_{\text{base}}^{\left( \mathcal{l} \right)} \right),$$

with $R=5$ repeats, and we also report the standard deviation across repeats as a stability indicator.

Interpretation:

- $\Delta_{j}^{\left( \mathcal{l} \right)}>0$: permuting feature $j$increases errors ⇒ the feature contributes positively to correct patient-level classification.
- $\Delta_{j}^{\left( \mathcal{l} \right)}\approx0$: little to no effect ⇒ the feature is not used (or its information is redundant).
- $\Delta_{j}^{\left( \mathcal{l} \right)}<0$: permuting improves error ⇒ the feature may be spurious/overfitting for that fold (rare but informative).

**Runtime control and consistency with feature selection**

To keep the computation tractable and aligned with the fitted pipeline, permutation importance is computed **only on candidate features that are actually available to the classifier**:

- If the pipeline contains SelectKBest, we permute only the subset selected in that fold for that label (retrieved via the selector support mask).
- We additionally cap the number of permuted features to the first $M=50$selected features per label per fold (configurable), to avoid quadratic runtime in $d$.

This is crucial: the importance scores quantify reliance on features **after** the selection stage used during training, not the importance of excluded features.

**Aggregation and reporting across folds / models**

The procedure yields a long-format importance table with, for each (model configuration, fold, label, feature):

- baseline patient error,
- $\Delta\mathrm{Err}$mean and standard deviation across repeats,
- feature identity (name, index),
- fold and model metadata.

Downstream summarization typically reports, for each phenotype, the top-ranked features (largest $\Delta\mathrm{Err}$) aggregated across folds (e.g., mean rank or mean $\Delta\mathrm{Err}$), and optionally stratified by feature families (statistical/temporal/spectral/complexity) and anatomical segments (landmark channels).

## Reference

1. Redmon J, Divvala S, Girshick R, Farhadi A. You Only Look Once: Unified, Real-Time Object Detection [Internet]. In: 2016 IEEE Conference on Computer Vision and Pattern Recognition (CVPR). Las Vegas, NV, USA: IEEE; 2016 p. 779–788.[cited 2025 July 28 ] Available from: http://ieeexplore.ieee.org/document/7780460/

2. Pedregosa F, Varoquaux G, Gramfort A, et al. Scikit-learn: Machine Learning in Python [Internet]. 2012;[cited 2025 July 28 ] Available from: https://arxiv.org/abs/1201.0490

3. Chen T, Guestrin C. XGBoost: A Scalable Tree Boosting System [Internet]. In: Proceedings of the 22nd ACM SIGKDD International Conference on Knowledge Discovery and Data Mining. San Francisco California USA: ACM; 2016 p. 785–794.[cited 2025 July 28 ] Available from: https://dl.acm.org/doi/10.1145/2939672.2939785

4. Breiman L. Random Forests. Mach. Learn. 2001;45(1):5–32.

5. Chawla NV, Bowyer KW, Hall LO, Kegelmeyer WP. SMOTE: Synthetic Minority Over-sampling Technique. J. Artif. Intell. Res. 2002;16:321–357.

6. Bradski G. The OpenCV Library. Dr Dobbs J. Softw. Tools 2000;

7. Cao Z, Hidalgo G, Simon T, et al. OpenPose: Realtime Multi-Person 2D Pose Estimation using Part Affinity Fields [Internet]. 2018;[cited 2025 July 30 ] Available from: https://arxiv.org/abs/1812.08008

8. Jocher G, Chaurasia A, Qiu J. Ultralytics yolov8. 2023. 2024;

9. Mathis A, Mamidanna P, Cury KM, et al. DeepLabCut: markerless pose estimation of user-defined body parts with deep learning. Nat. Neurosci. 2018;21(9):1281–1289.

10. Harris CR, Millman KJ, Van Der Walt SJ, et al. Array programming with NumPy. Nature 2020;585(7825):357–362.

11. Virtanen P, Gommers R, Oliphant TE, et al. SciPy 1.0: fundamental algorithms for scientific computing in Python. Nat. Methods 2020;17(3):261–272.

12. McKinney W. Data Structures for Statistical Computing in Python [Internet]. Austin, Texas: 2010 p. 56–61.[cited 2025 July 30 ] Available from: https://doi.curvenote.com/10.25080/Majora-92bf1922-00a

13. Bandt C, Pompe B. Permutation Entropy: A Natural Complexity Measure for Time Series. Phys. Rev. Lett. 2002;88(17):174102.

14. Bergstra J, Bengio Y. Random Search for Hyper-Parameter Optimization. J. Mach. Learn. Res. 2012;13(10):281–305.

15. Varoquaux G, Raamana PR, Engemann DA, et al. Assessing and tuning brain decoders: Cross-validation, caveats, and guidelines. NeuroImage 2017;145:166–179.

16. Sechidis K, Tsoumakas G, Vlahavas I. On the Stratification of Multi-label Data [Internet]. In: Gunopulos D, Hofmann T, Malerba D, Vazirgiannis M, editors. Machine Learning and Knowledge Discovery in Databases. Berlin, Heidelberg: Springer Berlin Heidelberg; 2011 p. 145–158.[cited 2025 July 30 ] Available from: http://link.springer.com/10.1007/978-3-642-23808-6_10

17. Niculescu-Mizil A, Caruana R. Predicting good probabilities with supervised learning [Internet]. In: Proceedings of the 22nd international conference on Machine learning - ICML ’05. Bonn, Germany: ACM Press; 2005 p. 625–632.[cited 2025 July 30 ] Available from: http://portal.acm.org/citation.cfm?doid=1102351.1102430

18. Vasques X. Machine learning theory and applications: hands-on use cases with Python on classical and quantum machines. Hoboken, New Jersey: Wiley; 2024.
