# Supplementary material for: Deep Learning Pose Estimation for Phenotyping of Co‐Occurring Hyperkinetic Movement Disorders
Source: Ann Clin Transl Neurol. 2026 Jul 25:10.1002/acn3.70474. Online ahead of print. doi: 10.1002/acn3.70474 (PMC13401409; doi:10.1002/acn3.70474)
Supplement: Supplementary file 4 — Table S1: Per‐phenotype window inventory and per‐fold class balance. [file ACN3-9999-0-s008.docx]

**Supplementary Table sT1. Per-phenotype window inventory and per-fold class balance.**

*Window-level annotations summarized per phenotype from the consensus-rater dataset used for the primary analysis (Laura Cif, LC). The window inventory (S2a) reports the total annotated 10-s windows, the number of positives (label = 1), negatives (label = 0), uncertain windows (label = 2), and mixed windows (i.e., windows that contained two or more distinct non-uncertain labels for the same phenotype across frames). Uncertain and mixed windows were excluded before model fitting; the "% excluded" column is the proportion excluded for each phenotype. The per-fold patient counts (S2b) show, for each of the 3 outer cross-validation folds, the number of participants with at least one window positive for the phenotype (P) and the number of participants with no positive window for that phenotype (N).*

**ST1a. Window inventory per phenotype (LC rater, all 25 participants).**

| **Phenotype** | **Annotated windows** | **Positive (1)** | **Negative (0)** | **Uncertain (2)** | **Mixed** | **% excluded** |
| --- | --- | --- | --- | --- | --- | --- |
| Dystonia | 2,710 | 1,842 | 703 | 103 | 62 | 6.1% |
| Tremor | 2,710 | 493 | 2,054 | 101 | 62 | 6.0% |
| Myoclonus | 2,710 | 168 | 2,409 | 100 | 33 | 4.9% |
| Chorea | 2,710 | 310 | 2,295 | 94 | 11 | 3.9% |
| Athetosis | 2,710 | 176 | 2,408 | 99 | 27 | 4.6% |
| Tics | 2,710 | 2 | 2,599 | 94 | 15 | 4.0% |
| Ballismus | 2,710 | 13 | 2,596 | 94 | 7 | 3.7% |
| Stereotypies | 2,710 | 62 | 2,487 | 94 | 67 | 5.9% |

**sT2b. Per-fold patient-level positive/negative counts (3 outer folds).**

| **Phenotype** | **Fold 1 (P / N)** | **Fold 2 (P / N)** | **Fold 3 (P / N)** | **Total P (participants)** | **Total N (participants)** |
| --- | --- | --- | --- | --- | --- |
| Dystonia | 7 / 1 | 6 / 2 | 8 / 1 | 21 | 4 |
| Tremor | 4 / 4 | 5 / 3 | 6 / 3 | 15 | 10 |
| Myoclonus | 5 / 3 | 5 / 3 | 5 / 4 | 15 | 10 |
| Chorea | 2 / 6 | 2 / 6 | 2 / 7 | 6 | 19 |
| Athetosis | 3 / 5 | 3 / 5 | 3 / 6 | 9 | 16 |
| Ballismus | 1 / 7 | 1 / 7 | 1 / 8 | 3 | 22 |
| Stereotypies | 3 / 5 | 2 / 6 | 2 / 7 | 7 | 18 |
| Tics | 1 / 7 | 1 / 7 | 1 / 8 | 3 | 22 |
