# Supplementary material for: Deep Learning Pose Estimation for Phenotyping of Co‐Occurring Hyperkinetic Movement Disorders
Source: Ann Clin Transl Neurol. 2026 Jul 25:10.1002/acn3.70474. Online ahead of print. doi: 10.1002/acn3.70474 (PMC13401409; doi:10.1002/acn3.70474)
Supplement: Supplementary file 5 — Table S2: Inter‐rater agreement (Cohen's κ) at the window level prior to consensus. [file ACN3-9999-0-s001.docx]

**Supplementary Table S2. Inter-rater agreement (Cohen's κ) at the window level prior to consensus.**

*Cohen's κ was computed at the 10-s window level between the two independent raters (LC and DD) before structured consensus discussion. Only windows for which both raters provided a non-uncertain, non-mixed binary label (0 or 1) are included in each per-phenotype computation. TN = both raters labelled the window as "phenotype absent"; 01 = LC absent, DD present; 10 = LC present, DD absent; TP = both raters labelled the window as "phenotype present". The raw agreement percentage (column 3) and κ (column 2) are reported together because κ can be heavily depressed by extreme prevalence imbalance even when raw agreement is high (the so-called prevalence-bias paradox); this is the case for ballismus, tics, and stereotypies, where the consensus prevalence is below 3% and both raters predominantly assigned the "absent" label. Stereotypies are the clearest example: among the 2,209 shared windows with clean binary labels from both raters, the first rater labelled 48 as positive while the second labelled none, so that κ = 0.000 despite 97.8% raw agreement, indicating that disagreement concentrates in the phenotypes for which the field still lacks shared operational criteria. κ values of 0.61–0.80 are conventionally described as "substantial", 0.41–0.60 as "moderate", 0.21–0.40 as "fair", and < 0.21 as "slight" or "poor". These thresholds are interpretive only.*

| **Phenotype** | **Cohen's κ** | **Agreement (%)** | **n shared windows** | **TN** | **01** | **10** | **TP** |
| --- | --- | --- | --- | --- | --- | --- | --- |
| Dystonia | 0.762 | 89.0% | 2,124 | 638 | 35 | 198 | 1,253 |
| Tremor | 0.724 | 92.1% | 2,190 | 1,722 | 66 | 108 | 294 |
| Chorea | 0.600 | 91.6% | 2,250 | 1,890 | 125 | 63 | 172 |
| Myoclonus | 0.366 | 93.4% | 2,220 | 2,024 | 55 | 92 | 49 |
| Athetosis | 0.259 | 92.3% | 2,239 | 2,030 | 41 | 132 | 36 |
| Ballismus | 0.220 | 99.4% | 2,255 | 2,239 | 3 | 11 | 2 |
| Tics | 0.116 | 99.3% | 2,247 | 2,231 | 14 | 1 | 1 |
| Stereotypies | 0.000 | 97.8% | 2,209 | 2,161 | 0 | 48 | 0 |
