# Supplementary material for: Deep Learning Pose Estimation for Phenotyping of Co‐Occurring Hyperkinetic Movement Disorders
Source: Ann Clin Transl Neurol. 2026 Jul 25:10.1002/acn3.70474. Online ahead of print. doi: 10.1002/acn3.70474 (PMC13401409; doi:10.1002/acn3.70474)
Supplement: Supplementary file 6 — Table S3a: Patient‐level sensitivity and specificity under post hoc per‐label best (exploratory upper bound). [file ACN3-9999-0-s004.docx]

**Supplementary Table sT3a. Patient-level sensitivity and specificity under post-hoc per-label best (exploratory upper bound).**

*For each phenotype, P = number of patients with the phenotype present (consensus label = 1), N = number of patients without the phenotype (consensus label = 0). Sensitivity = TP/P, Specificity = TN/N. 95% Wilson score confidence intervals are reported. This table corresponds to the exploratory post-hoc per-label best-pipeline analysis (Table 3 of the main manuscript) and yields the headline patient–label agreement of 172/200 = 86.0%. Because the pipeline minimising errors was chosen post-hoc per phenotype, these estimates should be interpreted as an upper bound on phenotype-specific performance achievable in this cohort, not as a confirmatory estimate.*

| **Phenotype** | **P** | **N** | **Sensitivity (95% Wilson CI)** | **Specificity (95% Wilson CI)** | **TP / FN** | **FP / TN** | **Errors** |
| --- | --- | --- | --- | --- | --- | --- | --- |
| Dystonia | 21 | 4 | 0.95 (0.77–0.99) | 0.75 (0.30–0.95) | 20 / 1 | 1 / 3 | 2 |
| Tremor | 15 | 10 | 0.73 (0.48–0.89) | 0.60 (0.31–0.83) | 11 / 4 | 4 / 6 | 8 |
| Myoclonus | 15 | 10 | 1.00 (0.80–1.00) | 0.60 (0.31–0.83) | 15 / 0 | 4 / 6 | 4 |
| Chorea | 6 | 19 | 1.00 (0.61–1.00) | 0.84 (0.62–0.94) | 6 / 0 | 3 / 16 | 3 |
| Athetosis | 9 | 16 | 0.78 (0.45–0.94) | 0.81 (0.57–0.93) | 7 / 2 | 3 / 13 | 5 |
| Ballismus | 3 | 22 | 0.33 (0.06–0.79) | 1.00 (0.85–1.00) | 1 / 2 | 0 / 22 | 2 |
| Stereotypies | 7 | 18 | 0.86 (0.49–0.97) | 0.83 (0.61–0.94) | 6 / 1 | 3 / 15 | 4 |
| Tics | 3 | 22 | 1.00 (0.44–1.00) | 1.00 (0.85–1.00) | 3 / 0 | 0 / 22 | 0 |
| TOTAL | 79 | 121 | — | — | — | — | 28 / 200 (14.0%) |
