# Supplementary material for: Deep Learning Pose Estimation for Phenotyping of Co‐Occurring Hyperkinetic Movement Disorders
Source: Ann Clin Transl Neurol. 2026 Jul 25:10.1002/acn3.70474. Online ahead of print. doi: 10.1002/acn3.70474 (PMC13401409; doi:10.1002/acn3.70474)
Supplement: Supplementary file 7 — Table S3b: Patient‐level sensitivity and specificity under nested cross‐validation (PRIMARY analysis). [file ACN3-9999-0-s003.docx]

**Supplementary Table sT3b. Patient-level sensitivity and specificity under nested cross-validation (PRIMARY analysis).**

*Same conventions as Supplementary Table S5a. The model and threshold for each phenotype were selected within training folds using nested cross-validation (no use of test-fold information). This corresponds to the prespecified primary analysis and yields patient–label agreement of 153/200 = 76.5%. The lower point estimates relative to the post-hoc upper bound (Table S5a), and the wider confidence intervals for low-prevalence phenotypes, reflect the absence of post-hoc model selection and the limited size of the cohort.*

| **Phenotype** | **P** | **N** | **Sensitivity (95% Wilson CI)** | **Specificity (95% Wilson CI)** | **TP / FN** | **FP / TN** | **Errors** |
| --- | --- | --- | --- | --- | --- | --- | --- |
| Dystonia | 21 | 4 | 0.90 (0.71–0.97) | 0.25 (0.05–0.70) | 19 / 2 | 3 / 1 | 5 |
| Tremor | 15 | 10 | 0.87 (0.62–0.96) | 0.30 (0.11–0.60) | 13 / 2 | 7 / 3 | 9 |
| Myoclonus | 15 | 10 | 0.67 (0.42–0.85) | 0.40 (0.17–0.69) | 10 / 5 | 6 / 4 | 11 |
| Chorea | 6 | 19 | 0.67 (0.30–0.90) | 0.79 (0.57–0.91) | 4 / 2 | 4 / 15 | 6 |
| Athetosis | 9 | 16 | 0.78 (0.45–0.94) | 0.75 (0.51–0.90) | 7 / 2 | 4 / 12 | 6 |
| Ballismus | 3 | 22 | 0.00 (0.00–0.56) | 1.00 (0.85–1.00) | 0 / 3 | 0 / 22 | 3 |
| Stereotypies | 7 | 18 | 0.43 (0.16–0.75) | 0.94 (0.74–0.99) | 3 / 4 | 1 / 17 | 5 |
| Tics | 3 | 22 | 0.67 (0.21–0.94) | 0.95 (0.78–0.99) | 2 / 1 | 1 / 21 | 2 |
| TOTAL | 79 | 121 | — | — | — | — | 47 / 200 (23.5%) |
