# Supplementary material for: Deep Learning Pose Estimation for Phenotyping of Co‐Occurring Hyperkinetic Movement Disorders
Source: Ann Clin Transl Neurol. 2026 Jul 25:10.1002/acn3.70474. Online ahead of print. doi: 10.1002/acn3.70474 (PMC13401409; doi:10.1002/acn3.70474)
Supplement: Supplementary file 8 — Table S4: Discrimination metrics with 95% bootstrap (BCa) confidence intervals across folds. [file ACN3-9999-0-s007.docx]

**Supplementary Table sT4. Discrimination metrics with 95% bootstrap (BCa) confidence intervals across folds.**

*Macro-average area under the precision–recall curve (macro-AUPRC, primary metric) and macro-average receiver operating characteristic area under the curve (macro-AUROC, supportive metric) under three model-selection strategies. Mean ± standard deviation is computed across the 3 outer cross-validation folds. Bias-corrected and accelerated (BCa) bootstrap 95% confidence intervals (n_resamples = 2,000) are reported as a measure of fold-level precision. NOTE: bootstrap CI bounds are presented as placeholders in this revision because they require an additional resampling step on the per-fold out-of-fold predictions; they will be inserted at typesetting from the publicly released code (GitHub repository).*

| **Strategy** | **macro-AUPRC (mean ± SD; 95% bootstrap BCa CI)** | **macro-AUROC (mean ± SD; 95% bootstrap BCa CI)** | **n outer folds** |
| --- | --- | --- | --- |
| Best single pipeline by macro-AUPRC (StandardScaler + MLP) : exploratory | 0.821 ± 0.019 (CI to be inserted) | 0.830 ± 0.029 (CI to be inserted) | 3 |
| Best single pipeline by Hamming accuracy (MinMaxScaler + SVM) : exploratory | 0.769 ± 0.049 (CI to be inserted) | 0.749 ± 0.094 (CI to be inserted) | 3 |
| Nested cross-validation, per-label best : PRIMARY | 0.717 ± 0.030 (CI to be inserted) | 0.767 ± 0.069 (CI to be inserted) | 3 |
